# Medicalizing risk: How experts and consumers manage uncertainty in genetic health testing

**DOI:** 10.1371/journal.pone.0270430

**Published:** 2022-08-04

**Authors:** Meghna Mukherjee, Margaret Eby, Skyler Wang, Armando Lara-Millán, Althea Maya Earle

**Affiliations:** Department of Sociology, University of California Berkeley, Berkeley, California, United States of America; Universita degli Studi di Roma Tor Vergata, ITALY

## Abstract

Given increased prevalence of direct-to-consumer (DTC) genetic health tests in recent years, this paper delves into discourses among researchers at professional genomics conferences and lay DTC genetic test users on popular discussion website Reddit to understand the contested value of genetic knowledge and its direct implications for health management. Harnessing ethnographic observations at five conferences and a text -analysis of 52 Reddit threads, we find both experts and lay patient-consumers navigate their own versions of “productive uncertainty.” Experts develop genetic technologies to legitimize unsettled genomics as medical knowledge and mobilize resources and products, while lay patient-consumers turn to Internet forums to gain clarity on knowledge gaps that help better manage their genetic risk states. By showing how the uncertain nature of genomics serves as a productive force placing both parties within a mutually cooperative cycle, we argue that experts and patient-consumers co-produce a form of relational medicalization that concretizes “risk” itself as a disease state.

## Introduction

Since the inception of the Human Genome Project (1990–2003), an endeavor to map the genetic composition of human DNA, medicine has undergone a dramatic reorientation. Medical advancements have steered increasingly toward personalized understandings of the human condition [1]. We refer to this as ‘personalized’ or ‘precision’ medicine, wherein genetic knowledge plays a partial yet indispensable role [2]. In addition to other transformations, personalized medicine uplifts the genomic scientist, who plays a critical role in developing technologies and influencing clinicians to implement products and analyses. It also brings democratized access to technology for lay patients [3, 4]. These factors enable increased participation in a medicalized worldview in which genetic biomarkers can inform understandings of our physical existence, health futures, and life choices. However, heightened technological insight does not always entail increased health certainty; uncertainty features prominently in the making of medicalization and continued ‘need’ for technological advancement [5].

Within the genomics revolution, direct-to-consumer (DTC) genetic technologies are transforming what it means to be ‘healthy.’ In “recreational genetics” [6], individuals can purchase a DNA kit for a modest sum, mail in a swab of their cheek cells (or another source of genetic data), and receive a detailed report of their genetic identity past, present, and future. Through augmented access to genetic data, consumers are often left with perceptions of ever-present health risk and must muddle through the unsettled terrain of genetic diagnostics and interventions [7]. Existing research explores how both lay and expert actors mobilize, often at odds, to medicalize or resist diagnostic conditions [8–10]. Research also establishes the multiple motivations behind actors’ medicalization of certain diagnoses, such as moralization, stigma, or strategic metrics [11–13]. Extending this scholarship, our paper considers how ‘risk’ is transformed into a health state within genomics. We question the increasingly common ways in which risk becomes an all-encompassing diagnosis in the personalized medicine era. When everyone presents with risk, and when health risks are determined prior to the onset of biophysical symptoms, what does ‘sickness’ or ‘patienthood’ entail? How do individuals make sense of this liminal and flexible health identity? Further, how does this ambiguity feedback into biomedical expertise and innovations?

We analyze discourses among researchers at professional genomics conferences and lay DTC genetic test users in Reddit patient communities to understand the contested value of genetic knowledge and its direct implications for health management. We argue that both groups cooperate in continually medicalizing risk as a diagnostic state, which has implications on lifestyle and health interventions. Genomics’ uncertain nature and the current limits of this science serve as a productive force in positioning lay and expert actors within a mutual feedback cycle [4]. Lay actors (or patient-consumers) seek medicalization to clarify how to manage their genetic risk, while experts who develop and advise on these technologies look to legitimate genomics as medical knowledge and further iterate products. Both groups leverage uncertainty and conceptualize risk as a health state that can (and perhaps should) be managed using perpetually ‘progressive’ genetic technologies. This strengthens the ethos of consumer-led health management, wherein individuals (as opposed to states or medical institutions) are behooved to manage their health and wellbeing [3, 5].

We underscore how medical uncertainty fundamentally shapes one’s diagnostic odyssey–the journey from diagnosis to intervention to resolution (or lack thereof) [14]. Borrowing from David Stark’s organizational account of uncertainty as a productive tool, we frame the genetic testing knowledge-scape as one that thrives off the same proliferation of multiple, discordant logics and unknowns [15]. Studies show how uncertainty operates in genetic testing (DTC and clinical), including disconnects between and among expert clinicians and patient-consumers [16, 17]. Where scholarship compares clinicians to patients, we spotlight genomics researchers–a relatively under-studied expert role. While physicians mediate clinical validity of genetic technologies, researchers develop analytically valid products that may find interest in the DTC health market. Analyzing researchers alongside patient-consumers, we find a distinct pathway for risk medicalization wherein experts and lay strategically act together [4].

Genetic health unknowns (and perhaps, unknowables) produce innovative approaches when conceptualizing one’s health status and future in a technology- reliant world. Though they do so differently, both researchers and patient-consumers strive to mitigate uncertainty surrounding genetic health tests while also leveraging this ambiguity to achieve disparate goals in their own right. Ultimately, both groups invest in uncertain genomic information–developing technologies or providing biodata and market interest–for the potential promise of clarity down the line. Given today’s technologically empowered patient-consumer and their abundant access to genetic data, it is increasingly important to uncover how medical innovations disrupt and re-envision the management of health, risk, and existence.

### The social construction of genetic health and diagnoses

What constitutes ‘healthy’ or ‘normal’ is far from stagnant or essentialized, bearing on how certain conditions become medicalized as diagnoses while others remain contested [18, 19]. Definitions of health evolve with augmented technological insight and sociohistorical politics contouring medicalization–the process by which “nonmedical problems become defined and treated as medical problems” [20, 21]. Importantly, intersections of gender, race, class, and cultural moralities shape how medicalization unfolds unevenly [8, 9, 11].

Genetic testing is a crucial site where health is constructed. Genetic sequencing, the technique grounding genetic tests, constitutes the body as “interchangeable parts, […] dismembering ‘physical health’ itself into separate territories to be divided and conquered” [1]. Making visible the granular components of our material selves–our DNA–these technologies amplify boundaries around normalcy and pathology, emphasizing health as an outcome of one’s genetic composition rather than structural circumstances [5, 22]. However, constructing genetic diagnoses can be challenging, given the complex ways in which one or more genes may be associated with a disease outcome. Genetic diagnoses are a moving target, wherein several (and many unknown) genes have the potential to ‘cause’ loosely defined medical outcomes [23]. Experts in genetic science also struggle to determine which external conditions are relevant to individual gene expression, which shapes provider-patient interactions [16, 17]. This shifting terrain leaves many patients in continued uncertainty and can weaken the significance of a medical diagnostic category [4]. Nevertheless, genetic diagnostic categories remain pertinent given patients’ demand for more precise interventions from healthcare providers and growing profits from the biotechnology industry [18].

Despite diagnostic uncertainty, sequencing advances have made consumer genetic testing increasingly affordable and accessible via private biomedical companies. The birth of this privatized marketplace fulfills “a drive to develop valid disease risk predictions and consequently offer tailor-made disease management and treatment,” which was left largely unaddressed in primary healthcare [24]. Consumer-targeted advertising for genetic products importantly frames diagnoses and risk, as marketers often overstate the clinical value of such testing. Companies may provide pseudoscientific misinformation, exaggerate consumers’ risks, endorse a deterministic relationship between genes and disease, and reinforce associations between diseases and ethnic groups [25, 26]. They echo genetic “reductionism and determinism,” wherein probabilistic genetic findings–the odds that a person may have a particular genetic condition–are inaccurately interpreted as absolute health fates [1, 6].

### An era of individualized risk responsibility

Scholars describe our contemporary health era as one that embraces the “increasingly technological and scientific nature of biomedicine;” is characterized by ongoing “elaboration of risk and surveillance;” and allows a radical diversification in how medical knowledge is “produced, distributed, and consumed” [3]. These transformations mark an increasingly individualized approach to managing health risk. Here, it is not public infrastructures that bear responsibility of population health and equity, but rather individuals who must exert resources to manage health and risk on a personal level. This approach moralizes individuals’ ethical duty toward consistently managing health risk, often using available technologies [5, 27, 28]. Importantly, genetic products promote this paradigm where health status is understood on the individual rather than structural level [1, 29].

There are several concerns with individualizing health management, especially via genetic testing. First, as discussed, genetic determinism is flawed given the complexity of interpreting genetic health, gene expression nuances, and epigenetic interactions [1, 22, 23]. Second, reducing health to genes (and risk scores) paves the way for a “new social underclass based on genetic discrimination,” “especially (via) the health and life insurance industries,” where individuals may be stigmatized based on genetic findings, many of which may never manifest [30]. Third, in addition to ethical, legal, and regulatory vacuums [31], consumer-based genetic tests, many of which lack “clinical utility,” could traverse into built healthcare systems and sway primary health management [24]. Without robust accessible public healthcare, genetic tests present an opportunity to illuminate one’s health risks and reconcile uncertainty about health futures, while reinforcing the ‘responsibility’ to privately manage health using genetic information.

### Contested expertise and the production of uncertainty

Medical knowledge production is multi-dimensional and often created outside laboratories, hospitals, and medical schools. Key non-medical actors include health advocates, political stakeholders, private industries, and patients themselves, all of who contribute to how diseases and conditions are framed and socially prioritized (or neglected) [8, 9, 11, 12]. Often, there is contestation between lay and expert stakeholders, as lay actors resist expert framings or experts disregard lay health experiences [10, 32, 33]. As such, medical expertise is co-created among networks of actors, institutions, and technologies, where alternate expertise continually emerges [34].

Turning to genomics, we see collaboration and contestation among lay and expert actors. Alongside physicians, ‘expert’ actors include genetic counselors and genomics scientists. Timmermans and Stivers show the divergent ways patients and genetic counselors make sense of genetic information, noting discrepancies in interpreting results and making subsequent life decisions [16]. On the other hand, patients and geneticists collaboratively interpret findings, reconciling “genetic causality” with inherent “diagnostic uncertainty” in test results [16]. Navon and Eyal [4] and Navon [23] center on patient advocates and genomics researchers, showing how these groups cooperatively–though for separate self-interests–create and expand diagnostic categories and treatments based on evolving genetic mutations and variants. Such work illuminates not only how genomic uncertainty can be especially productive among expert and lay actors, but also how genetics data only ’makes sense’ during interpretive interactions between different stakeholders–a process best captured through ethnographic methods. Furthermore, where many studies focus on expert-lay contestations, the consideration of non-clinician experts–like genomics researchers–suggests a pathway for medicalization grounded in cooperation alongside contestation.

The private industry around DTC genetic testing also impacts genetic knowledge. Patient-consumers seek DTC genetic services to glean health risks, inform lifestyle modifications, and satisfy curiosity about genetic futures [35]. However, because DTC companies like 23andMe tend to “blackbox their representation of predicted risks,” test results “only become meaningful through individual users’ continuous interpretive work upon the data,” e.g., by sharing and discussing it with others [36]. As such, patient-consumers often turn to open-source information on the Internet and market messaging around health and medicine. Online, patient-consumers support one another in obtaining consumer genetic tests, interpreting results, and informing subsequent health decisions for themselves and family members. These self-help communities co-construct an “illness identity” and justify subsequent decisions for one another [32]. Unsurprisingly, those patient-consumers who can effectively participate in these communities are socioeconomically privileged, scientifically literate, and savvy Internet users [37, 38].

Augmented access to medical information, however, does not mean patients accurately understand their health states [32, 39]. Studies show that individuals’ understanding of genetic data on online forums differs significantly from experts’ [40, 41], and that patients may experience unnecessary concern, false reassurance, or unwarranted changes in health behaviors [42, 43]. Patients have limited support interpreting DTC genetic results. A recent national study showed that 42% of physicians surveyed had been asked by a patient about a DTC genetic test, and 15% were asked to help interpret these results [38]; whereas, only around half of clinical geneticists could assist with unpacking DTC findings [44]. Further, because DTC products have “limited predictive value for consumers” [31], many become “patients-in-waiting”–suspended in limbo because their diagnosis and its implications cannot be scientifically confirmed [14]. As consumers navigate health uncertainties, they make interventions informed by probabilistic risk-scores, and such as, turn risk itself into a destabilizing state necessitating ‘treatment.’

Aside from a 2007 American Society of Human Genetics statement discrediting the medical utility of DTC genetic testing and urging more regulation over companies, consumers have little official guidance on navigating this marketplace [45]. With loose regulations, private companies can purport pseudo-medical claims and a “more serious, medically and scientifically reliable appearance to attract online shoppers” [46]. Accordingly, it is worth asking how expert and lay actors interpret this rapidly developing yet persistently uncertain space of genomic information, and how their knowledge-making strategies complement or contend with one another.

## Methods

This study utilized a multi-method approach over a two-year period (2018–2020). Relying on established offline and online ethnographic methods commonly deployed in the sociology of health and medicine [32, 47]. We employed participant observation or ethnographic observation to collect data on experts’ knowledge-making, and web content analysis to evaluate experiences of patient-consumers.

Ethnographic observation is particularly useful for understanding the medicalization of genetic information for two reasons. First, because genetic information is fraught with medical uncertainty (both in its clinical relevance and etiological basis), sociologists have shown that what makes genetic information useful or “actionable” to both physicians and patients is by situating such information in the social contexts of patients’ lives and the clinical contexts of researchers lives [16, 17]. It is in discussing how the information is relevant to particular contexts that patients and their physicians come to understand how the information can be useful for medical decision-making. Second, sociologists have shown that humans create meaning through interaction and ethnographic observation is an important method for studying interaction [48, 49]. That is, the meaning of a genetic test result and how it is clinical relevant is something that is created iteratively as patients interact with family, friends, and physicians [17]. The study was approved by the Committee for Protection of Human Subjects (CPHS) at the University of California, Berkeley (#2020-01-12894). CPHS did not require the research team to obtain consent because data collected was considered "public" data and was collected anonymously.

Our ethnography included five major genetics conferences across the U.S. during Spring 2020 (Table 1). We selected conferences that had a stable presence in the field (organized at least twice before), attracted a national audience, and engaged both medical practitioners and industry leaders in genetics. To ensure we collected comparable and accurate data about the state of genetic testing, we attended conferences occurring within three months of each other. Although these conferences target a variety of precision health issues, they all highlighted the growing importance of genetic testing in patient care, from the physicians’ offices to pharmaceutical manufacturers and laboratories. Some of these sessions included topics like quantitative genetics, genetic engineering, biobanks, precision medicine, phenotyping, gene regulation, and disease modeling.

**Table 1:** Summary of conferences attended to observe expert knowledge-making.

Conference	Location	Target Audience
**The Allied Genetics Conference (TAGC) hosted by the Genetics Society of America**	National Harbor, MD (virtual)	Genetics researchers at academic institutions and private companies, graduate students in the sciences
**4th Annual Columbia Precision Medicine Initiative (CPMI) conference: Advances in Precision Medicine: Harmonizing Clinical and Genomic Data**	New York, NY (virtual)	Academics and researchers in the genetic sciences, medical professionals
**Molecular Medicine Tri-Conference: Precision Health**	San Francisco, CA	Pharmaceutical representatives, industry leaders and researchers, business analysts, marketing associates, few academic researchers
**Molecular Medicine Tri-Conference: Digital Health**	San Francisco, CA	Pharmaceutical representatives, industry leaders and researchers, business analysts, marketing associates, few academic researchers
**Molecular Medicine Tri-Conference: Bio- IT World**	San Francisco, CA	Pharmaceutical representatives, industry leaders and researchers, business analysts, marketing associates, few academic researchers

We attended three conferences in person but participated in the rest virtually due to the COVID-19 pandemic. At least two researchers attended each conference and observed attendee presentations and interactions (virtual interactions took place on Slack or Zoom chat). Further, at least two researchers gathered data on each presentation panel to ensure consistency and reliability. In addition, we could revisit virtual conference recordings for clarification and comprehensiveness. We analyzed the data as a team. The codebook, a mix of theory and emergent themes from data, identified how scientific experts purported links between genotype and phenotype, ratio between questions and answers, scientific puzzles and hopes for solutions, genes posited as health markers, and degree of collaboration across fields and agendas.

To gather data on lay experiences of DTC genetic testing and the diagnostic odyssey, we selected and coded relevant Reddit threads. Reddit is a popular online platform where users can create and engage with communities and discuss a wide range of topics. Founded in 2005, Reddit is the 7th most visited website in the U.S. and the 17th most visited in the world. As a digital forum where people often engage frankly, the platform holds rich text-based data. Reddit’s popularity, combined with its community emphasis on information sharing, makes it an excellent site for interactional content-analysis (similar to platforms such as Twitter; [47]). Other studies utilizing digital ethnographies of Reddit to make sense of users’ interpretive processes have honed in on issues relating to mental health [50], the intersections of genetic testing and race [41], and a variety of chronic diseases [51]. One of the distinctive advantages of using data from websites like Reddit to understand changing health landscapes is that it allows researchers to capture the fast-changing nature of biomedical cultures and evaluate how such processes unfold via collective sense-making. Previous studies of unsettled patient status designation have emphasized interactions within patient groups to demonstrate the importance of peer support in navigating socio-medical spaces [52].

To build our Reddit dataset, we first assessed a sample genetics report provided to users of 23andme, the most popular and influential DTC genetic testing company in the U.S., to determine the health risks and conditions being tested for 23andme is the only DTC genetic testing company to receive marketing authorization by the FDA in four health categories: genetic health risk, carrier screening, pharmacogenetics and cancer predisposition [53]. Patient-consumers using 23andme can receive over 150 reports based on their genetic material, which is categorized into Health Predispositions, Carrier Reports, Wellness Reports, and Trait Reports. We then identified subreddits (topic-dedicated forums) that discussed health-related 23andme test results. In order to optimize for threads that showcased sustained interaction, which is essential to how individuals make sense of genetic data, we filtered subreddits based on their active users (50 thousand or more) and relevant discussions (keyword search for terms like “genetic test,” “gene,” “23andme,” “diabetes,” “cancer,” and 23 other disease states derived from the genetic reports aforementioned). With a list of relevant subreddits and keywords, we built a Python program that scraped each of the subreddits for threads that included at least one of the keywords. The program outputted all threads, their original post, and responses. We manually evaluated threads to ensure relevancy before analyzing. Our Reddit data consisted of 52 threads representing interactional exchanges around DTC genetic testing and results concerning 25 disease states.

One researcher coded each Reddit thread, which was then analyzed and reviewed collectively to resolve any discrepancies. This codebook represents existing scholarship and emergent ideas from the data. Codes overall reflect interactions about health states and futures based on genetic information, including the diagnostic odyssey, emotionality, perceptions of test reliability and purposes, uncertainty management, and life interventions.

While conferences and Reddit threads represent differing avenues and approaches to comprehending genetic information, both yield critical insight into persisting knowledge gaps. For Reddit users, individualized risk responsibility, as well as possibly resolving genetic health uncertainty, ensures buy-in to medical knowledge co-production. Genomic experts, eager to capitalize on the next wave of biomedical advancement and respond to demand for personalized treatments, simultaneously create idealized prospects for genetic testing even as they contend with limitations of current science.

## Findings

### Experts

#### The present uncertainty and idealized future of precision medicine

For experts across conferences, it was uncertainty about the scientific basis of precision medicine that was leveraged strategically to elevate genetic testing’s efficacy and importance to health in the future. Specifically, accounts of potential breakthroughs in genetic health were centered around current diagnostic uncertainty and limits of clinical data on disease phenotypes. By leveraging uncertainty, experts were able to underscore the need for better data and highlight the institutional barriers preventing genomics from reaching its maximum potential. It was the limitations of science–not its correctness–that was invoked to solicit resources and bolster investments in the field.

A stark example of the way that uncertainty is positively deployed are discussion of market opportunities at scientific conferences. Although many scientists acknowledge the unsettled nature of genomics, little stops products from being released into the market and medicalizing risk. Furthermore, rapid entries into the market engender supplementary services and cursory products designed to ameliorate uncertainty surrounding the first batch of products. For example, after acknowledging that physicians struggle to interpret genetic data, a software company presented their latest product aimed at tackling this issue. Moreover, presentations emphasized that because precision medicine remains a nascent field, mistakes and errors should be expected (and forgiven).

To illustrate uncertainty being leveraged, a conference tour is instructive. After briefly reviewing the genetics revolution, a keynote speaker at a Molecular Medicine Tri-Conference told an anecdotal story that caught the attention of many during a heavily attended plenary session. The story–which was awarded a Pulitzer Prize in journalism–was about an effort by doctors using genetic screening to diagnose a young boy’s mystery illness. Before the geneticist got to him, the boy was near death, and his doctors were resorting to drastic measures (i.e., immune suppressants, removing intestines). The speaker regaled the audience of scientists:

There was no code. In two months, we worked weekends, middle of the night, to write the software to analyze his [genetic] data. One evening we identified the [genetic] variant… we put it on a list of ten possible culprits and had to meet with the immunologists to eliminate them one by one. Much wasn’t known about this variant and a paper happened to come out that connected it to a pathway. With that, we finally could convince the immunologist to confirm with tests that he was suffering from the rare disease. Nic is now 15 and he’s doing reasonably well with the missing intestine and does suffer from PTSD. He was in pain for a long time and was away from his family. We could have done a lot better if we had just sequenced [the boy] when it first showed up.

The anecdote (and the audience’s positive reception) encapsulates the idealized future of precision medicine. It evinces the field’s public-facing frame, as experts offer a hopeful narrative of genomics’ heroism in the face of medical uncertainty. Somewhat emblematic of a social movement within healthcare, precision medicine researchers state their objective as ending patients’ diagnostic odysseys using genetic testing—a future wherein patients would present doctors their entire sequenced genome. As another researcher said during their presentation at this conference, “The idea is to get the right patient, the right drug, at the right dose, at the right time.”

Similarly, at The Allied Genetics Conference (TAGC) put on by the Genetics Society of America, one speaker looked excitedly towards a future where “most humans will have their genomes sequenced” as scientific discoveries become both more human-focused and more industry-linked. In his vision, with the best of academia and industry, we should be able to scale genetic research rapidly and ultimately understand and cater to specific genetic needs of “every single person on the planet.” In stressing a more genomic-centric future, experts emphasize the uncertain state we currently live in (and need to move away from). These framings of uncertainty strategically spotlight turning unsettled science into a settled one.

Throughout the conferences, medicine researchers underscore one specific challenge to the idealized future: the field could solve more health problems if only it had more access to patient data. Many presentations referred to the linking of electronic health records–with their detailed medical coding schemes and physician descriptions of patients–to laboratory data. Participants suggested linking these sources with data that captures individuals’ social determinants of health, such as geo-tracking and wearable devices. Additionally, researchers emphasized the need to get genetic data from people of color, though they recognized the challenges with doing so given systemic experiences of medical trauma. With such data, they posited that machine learning could definitively link genetic variation to phenotypic disease expressions (and, not incidentally, rule out social determinants).

Uncertainty within this public frame is about the availability of data, not underlying methods of measuring and analyzing it (though many participants posed questions critiquing presenters’ methods). That is, if only researchers could get access to enough data, they could establish links between genetic variation and phenotypic diseases that inform treatments for patients. There is little mention of the downsides to medicalizing risk. It is common for conference presenters to fawn over the willingness of health systems to share large EHR datasets, especially those reaching far back in time. This corroborated the underlying principle of precision medicine seeking data to justify its idealized ends (and present uncertainty); as one presenter said, “For people in our genetics clinic … what the patient population can benefit from are these… rare disease patients are all unique and can have the same variations but different clinical paths for outcomes. If I can get the tickets of the patients by identifying the modifier, I can get them a few more years of health. And maybe the tech will help them … hopefully the rare becomes the chronic.”

#### The flipside of the coin

This idealized future of precision medicine is contrasted with experts’ strong understanding of current scientific limitations. Such complications include, but are not limited to: genetic variation only explains a small fraction of phenotypic disease, *denovo* variation occurs during the life course, social determinants are hard to measure, medical coding practices vary across health systems, researchers disagree over the best statistical models to link disease to genetic variation, and most importantly, practicing physicians are unclear on how this knowledge informs clinical decision-making.

A poignant example comes from one presenter who launched his talk with claims that within the next ten years geneticists will have identified nearly all genetic variants causing human disease to, later admitting during his talk the great challenges in understanding how variations contribute to disease and how genetic risk translates into treatments. He noted, “We really don’t know a lot about many of the genes in humans” and pointed to the fact that half of the papers published are on 5% of known genes. Thus, his positivity was less a promise and more a call to action to transform this genetic dataset into ‘real’ medical advancements. The unsettled science proliferates into each presentation and subsequent question and answer periods (Q&A) and is collectively brainstormed by researchers as something they must resolve.

The tenuous link between genetic variation and phenotypic disease expression is a central discussion at precision medicine conferences. Scientists widely acknowledged difficulties around identifying a genetic variation of possible interest. In one example, a researcher described how, previously, they would assume a particular variation was too messy and it would thus go ignored until they discovered the same variation could be layered amidst normal genetic groupings. Acknowledging the evolving science, the researcher said, “It will be much easier in the future.” In one Q&A session, an audience member asked what a presenter’s sense of the human genome’s dynamism was and how much it varied over a person’s lifetime. “It’s an obscure phenomenon,” responded the presenter, “it happens much more in complicated variations that we know of. We are seeing some (variations) override others at certain points, and then that one will override it back.” Another speaker noted, in humans, the ‘noise’ caused by myriad gene-environment interactions–whether that be diet, multiple disease states, or something as simple as aspirin—results in the admission that “the power to detect these interactions is very low.” One scientist dramatically said that “God’s punishment for geneticists’ hubris” was reflected in how a single genotype may influence multiple seemingly unrelated phenotypic traits. These technical negotiations show that scientists are grappling with genetic variation and its linkage with disease.

Another major issue pertains to how genetic data is analyzed. Human genetic code is too complex for current computers to adequately analyze its entirety. Instead, computers use algorithms to sample and predict portions of the code. Choosing how best to do this engenders great consternation among researchers. Discussing a $100 genetic sequencing in China, a presenter said, “Every method has its artifacts and problems. At some point, you start to say to yourself: why would I convert? Since they all have their problems … There is a base and then they come in later with a model that is more accurate.” Another researcher spent five minutes of a twelve-minute presentation discussing a “consensus approach” to choose from four algorithms in their analysis.

Electronic health records have their problems for researchers too. Local coding practices is one major hurdle. Medical codes that represent diseases and treatments are a key for how precision medicine tries to link phenotypic expressions with genetic variation. But what if hospitals and physicians’ offices practice coding differently? Pointing to ‘too much variation’ within hospital systems in one city that made EHR data available, a researcher described manually translating all data into a common language. Because EHR records were not designed for research, interpretability of phenotypes in medical databases is limited by the subjectivity of medical terminology, lack of standardization, and error, resulting in extreme difficulty extracting relevant concepts from records.

Unsettled science within precision medicine results in two practices: focus on single-gene variations to demonstrate scientific utility, and efforts to obtain buy-in from physicians. Despite the public-facing idealized future that more data on variation will explain diseases, most conference papers focus on unraveling a single, highly visible disease. Usually, after highlighting the robustness of their dataset, presenters focused on one disease. One presenter said, “This is nation-wide data. We were funded to investigate macular degeneration. It is the leading cause of adult-onset blindness. Diagnosis is made through imaging. It has a sizable genetic component … Two variants account for about 20% of the risk.” The researchers said this “unusual” situation in which so much of the disease (20%) could be predicted by genetic variation was a good reason to study it. This conveys the widespread nature of unsettled genomic science: there are enough controversies in measuring genetic variation and disease linkages that research is valued even if it works to solve only highly visible examples of its variants.

Physicians are seen on a continuum of potential allies to a major obstacle in the precision medicine movement. There is frustration around physicians having little knowledge about translating genetic risk to clinical action. In response to an audience member asking what the biggest challenge facing precision medicine is, a presenter answered:

The medical workforce is entirely unprepared for the genomic revolution. How do we upscale regular working physicians? So they can know enough to save children’s lives! … Most physicians have never seen these rare diseases before; they have no frame of reference. That their patients would benefit from a treatment regimen … We want NICU teams to practice this. In the next five years, it will be routine to do it. It gets better all the time. The biggest problem is physicians knowing about this. We need to convince people that this is solid, share the information, and build this.

Another participant spoke about developing a software platform to educate physicians to use genetic data. The platform was marketed to physicians as an easy way to provide “evidence-based” knowledge utilizing genetics for clinical decision-making. Importantly, these efforts and frustration with lack of physician buy-in co-exist alongside acknowledging that scientists themselves struggle to find clinical relevance for genetic information. Here, uncertainty is clearly leveraged–by bringing a product to clinicians, genetics companies instill a sense of legitimacy that the product may have productive potential in the future. The unsettledness of the backstage motivates the forward-facing science that appears to be certain and ready for application. The call for further integration of genome sequencing into routine medicine is based on the hope that alongside integration will be a demonstration of clinical utility and an implicit legitimization of genomic medical science. However, what makes a genetic risk result medically actionable and what percentage of patients will benefit from the medicalization of risk is left vague.

Genetic counselors play an ambiguous role in this discussion. At some junctures, conference participants lamented physicians as a lost cause and focused on increasing patients’ access to genetic counselors. As one presenter discussed, “There is an inequality to this … we lack the analysis part. It is direct-to-consumer now. We need to make the analysis of it better.” There may be an imminent professional battle over the control and authority of genetic data. Who should professionally analyze it—physicians, genetic counselors, or patients? Per precision medicine researchers, it matters little so long as products are brought to market fast and at scale.

To synthesize, it is through ethnographic observation that we are able to uncover the link between the public facing rhetoric and private consternation of precision medicine scientists. While conversations amongst scientists may signal important aspirational rhetoric as well as the need for scientific caution, highlighting key scenes at conferences allows us to understand the co-construction of meaning during interaction. In this section we showed that precision medicine scientists highlight the uncertainty in the field as a justification for continued investment (access to new and better data) while spending significant time trying to overcome serious scientific controversies (problems in sampling, data analysis, variations in data collection methods etc). These observations of presentations, audience feedback, applause, and shared themes across the conferences illustrate the degree to which leveraging uncertainty characterizes the scientific field of precision medicine.

### Patient-consumers

While experts utilize uncertainty to project optimistic futures, accrue resources, and gain market traction, DTC testers represent the consumer side of the same equation. Consumers inherit these experts’ unsettled science and attempt to turn ambiguous diagnostics into useful health directives. Embarking on diagnostic odysseys, patient-consumers of DTC genetic tests assume a status identity—a ‘responsible’ individual assuming control over their health decisions and destinies. However, like experts, resources for patient-consumers fall short of allowing them to realize their goal. With few interpretive tools and general skepticism, consumers seek insights and affirmation from online communities to fill knowledge gaps within genetic test reports.

The information patient-consumers use to fill these gaps come from a variety of sources ranging from anecdotal to professional. On Reddit, users (or posters) post questions or topics of discussion, which other users engage with through written replies and other indications of opinion (up-votes for posts they like, down-votes when they disagree). Unsettled genomic science leaves room for interpretation—online forums become important sites of contestation and collaboration as users share and compare framings for patient status/disease states [9].

#### Shaky science

Original posters (OPs) on threads begin with a level of uncertainty. Although they provide varying degrees of knowledge of their diagnosis, nearly all posters engage in the virtual community to triangulate and “cobble together” a sufficient body of knowledge to feel more secure in their understanding of their condition. These are typically conditions that have come to light during a genetic test or within one’s family medical history. In cases where a person has taken a genetic test, the typical post begins with some information about their results, a statement about their personal or family background, questions they have for the community, and sometimes solutions or pathways they are contemplating. In other instances, posters may ask whether others in the Reddit community recommend taking a genetic test for a particular condition, and a similar discussion ensues based on others sharing test results and interpretations.

In one case, OP, who admitted he does not know “a lot about health sequencing,” took a genetic test and was “shocked” that his results showed an increased risk of age-related macular degeneration. He wanted to know if he should take the results to a specialist and if the specialist might laugh at him or help him decrease his disease chances. His post highlights a trend that pervades Reddit threads–testers often do not know if medical experts take consumer-driven genetic testing seriously. This imbalance in acceptance and use of genetic testing across the medical profession might explain one of many motivations behind why many turn to Reddit forums. In contrast to the previous section in which an expert described understanding genetically-induced macular degeneration as developing and unsettled, patient-consumers interpreted an increased risk for macular degeneration as a certain diagnosis, eliciting strong emotional responses despite the uncertain nature of their case.

In most cases, however, subsequent posters recommend seeking medical attention to verify results. Ultimately, the medical institution, be it via physicians or genetic counselors, is invoked as the arbiter of legitimacy on genetic health (not DTC testing results). Here we see a central tension in individualized health management—at the end of the day, our ability to control our bodies and certain disease states is still dependent on medical experts and institutions. Many genetic hobbyists recognize the ‘recreational’ or ‘incomplete’ aspects of consumer-driven genetic health testing. For example, in response to an OP’s post on finding out that they don’t have a BRCA gene despite their “dad getting breast cancer,” a poster replied with:

23andMe is NOT the same as getting BRCA testing through a medical lab. They only check 3 out of thousands of possible mutations in the BRCA genes (and these 3 are only relevant if you have Jewish ancestry).

Another poster added:

Please consider using proper medical care to evaluate this situation, and not rely on

23andMe—It is NOT a medical test despite their ambiguous marketing.

Above, we see a clear hierarchy that pits DTC genetic tests against more “legitimate” tests. Many times, recipients of worrying results from companies such as 23andme are directed to more formal lab examinations to ascertain the odds of them having a particular disease or health risk.

In another example about perceived uncertainty or unreliability of 23&Me tests, an OP was encouraged to pursue additional testing even though “it was unlikely that [they] had both genes for MCADD (a rare genetic condition limiting a person’s ability to break down fat in their body) due to the low accuracy of 23andme.” The OP further notes, “the genetic counselor basically told me that 23andme is pretty notorious for false positive results.” Subsequently, some posters also challenge the test’s legitimacy by noting the FDA’s actions to control and regulate this industry. One poster mentions “The FDA came down on 23andMe really hard for providing genetic analysis of data they provide without a doctor’s prescription.” The poster refers to an ongoing FDA attempt to regulate DTC genetic testing and better inform the public about what these technologies can (but mostly, cannot) say about one’s health [54]. Generally, these threads present a common strategy of framing DTC genetic tests as perhaps useful consumer-health tools, but ones that are fundamentally inferior to genomic medicine that emerges from biomedical institutes. They cast doubt on DTC genetic testing using the stances presented by biomedical institutional authorities, reifying the boundary between pseudo- or para-medical DTC genetic testing and more ‘legitimate’ sources of medical knowledge. In other words, we observe that in navigating scientific uncertainties passed down by experts, patient-consumers create and expand sites of contestation through co-construction to gain a sense of autonomy and productivity that reinforces their self-responsible identity.

#### Acting on results

Despite significant evidence pointed at the shaky science behind DTC health testing, posts most commonly focus on sharing knowledge on lifestyle interventions in response to genetic test results (i.e., suggesting diet changes, healthy eating, and exercise). This contradiction is crucial to highlighting how much genetic insight has altered our conception of health. Even though Reddit users frequently raise doubt regarding DTC tests’ reliability and urge people to get ‘proper’ verification from medical institutions, many simultaneously advocate for lifestyle changes that suggest, to a certain degree, that one should take DTC test results seriously.

Because those who frequent these forums tend to have little or no medical background, lifestyle interventions are generic or low-stakes solutions that most feel equipped to offer. In other words, lifestyle interventions appear to be a safe and accessible approach to providing a poster with some reprieve while also allowing users to participate in discussions without bearing too much responsibility for another person’s health outcomes. Furthermore, lifestyle changes may also be a low-cost way to mitigate anxiety associated with medical uncertainty. Several users echoed this sentiment: “While I am waiting for verification, why not try to improve my health?” Although this medical management strategy is not as promising for those with dire or untreatable diseases, for many, it is a coping mechanism that gives a sense of control during times of uncertainty.

Recall the OP who posted about their health risk of age-related macular degeneration. In the thread, there were discussions that the odds of him getting the disease could be better ascertained by performing additional testing through more verified genomic medicine services like Promethease and ClinVar. The OP, however, reflects an awareness of the multifaceted causality of such a health condition and adds nuance to the perception of risk as a medical state. He notes, “It is usually just different probabilities. Most things are at least somewhat environmental. The advice is similar to what we’ve always heard. Eat your vegetables, watch your weight, exercise, and don’t smoke.” He understands that genetic insight can only say so much and underscores external factors, such as environmental and structural conditions and lifestyle choices, as modes of medical uncertainty management.

In another thread discussing results around Alzheimer’s and Parkinson’s disease, one poster mentioned:

I didn’t do the health report but I ran my raw data through Promethease. I have one copy of the Alzheimer’s gene, APOE4, which doubles my risk. My grandpa had Alzheimer’s so I wasn’t surprised. I’m glad I know this because there is research showing that E4 carriers can significantly lower their risk with exercise and with DHA supplementation, that saturated fat seems to be a unique risk factor, and that a more Mediterranean style diet may be protective. If I hadn’t have known (sic), I wouldn’t have been as likely to make some dietary and lifestyle changes.

Again, we see how genetic insight can spark low-burden lifestyle changes, which act both as a direct response to test results and enable patients-in-waiting to actively cope with their ongoing health states. In rare cases, DTC results led to an immediate change in individuals’ existing medical treatments: “The one good thing that came out of taking the test was that I was able to make some better-informed choices about my medications. Knowing that I have an increased risk of alzhiemers (sic), I talked to my doctor about getting off of a medication that has been associated with alzhiemers (sic). It hasn’t been confirmed yet, but given my genetic risk I no longer want to take any chances.” It appears that DTC genetic tests, as uncertain as they may be, reify health risks and motivate behavioral changes. Reflecting the medicalization of risk, consumers assume the role of patients even in the absence of concrete medical confirmation and commit to health and lifestyle interventions as a result of risk-based information. Such findings indicate that DTC tests not only have direct implications on people’s health management strategies, but they provide an important data point for clinicians, researchers, and biotech companies to understand how such strategies are mediated through how individuals make sense of their health data outside institutionalized medical purview.

#### ‘Experts’ on Reddit

Less frequently, expert opinions from medical or genetic professionals enter the forum, either directly from Redditors who are professionals in these fields or through second-hand diagnostic relays from lay Redditors. Experts’ posts carry extra weight on these forums in terms of their credibility. Their advice is most effective at minimizing user uncertainty because it represents a highly informed viewpoint, per institutional standards. In one popular thread, a Ph.D. researcher who developed a system to help diagnose celiac disease hosted an “Ask Me Anything” (AMA) event for other Redditors. This thread reveals the uncertainty around diagnosing celiac disease in the medical field. In particular, OP emphasizes that celiac disease might not be entirely genetic:

We need to be clear [that] having the [celiac] gene[s] does not mean one has celiac. One dataset reported over 30% of the non-celiac disease population has the genes but not the disease.

While agreeing that a genetic test is a reliable means of confirming a professional diagnosis of celiac disease, this expert OP also noted the dangers of relying on DTC testing, or what they call “internet tests,” and using symptoms to self-diagnose. Here, we see a re-emphasis on ‘legitimate’ biomedical knowledge and distinguishing DTC kits as sub-par to that standard:

If done the proper way the genetic test is highly reliable. There are some odd internet test kits that I have discovered were inaccurate once I ran the sample through a licensed laboratory. Please also note, and I can’t stress this enough, non-celiac gluten intolerance is real and presents with very similar symptomatology.

In another thread, an OP writes asking if a popular DTC genetic testing kit would address his concerns about a predisposition to heart attacks:

Hi guys recently found out some family members have had heart attacks on my biological dads side. (adopted by my actual dad) He says they have a faulty gene called FH or familial hypercholesterolemia. I was wondering if that came up on the 23 and me kit?

A self-professed medical professional responds to the post, cautioning against relying too much on genetic markers for a condition that can be easily diagnosed and treated regardless of genetic makeup:

The easiest way to find out if you have the severe genetic type of high cholesterol is to have your doctor check your cholesterol and lipid levels!

And you(sic) insurance will pay for it!

And if it is high, your doctor can treat it to reduce your risk of heart attack! Your doctor can’t treat with the DNA result. (Doctor and DNA tester here).

In this case, the professional diverts the OP’s interpretation from an overly geneticized health conceptualization to one that relies on other physiological markers.

The experts posting on Reddit embody the uncertainty that medical knowledge production across various spaces creates. Outside of a clinical setting, and without any way to assert (or prove) their identities as medical experts, they join the melee of posters whose advice runs from dietary changes to ethical reasons for forgoing reproduction. While these experts are joined by many lay posters encouraging a more systematic approach to diagnosis and treatment, they are also left somewhat adrift by individuals’ new and evolving predisposition to geneticize healthiness. In one thread begun by a poster titled “Got the new type 2 diabetes results and they’re not good. Basically I have a 50/50 shot of getting diabetes?”, a self-described former nurse encourages a cautious read of these results:

If your lifestyle (diet & exercise) is the same as the research participants, yes. That may have been a small number of participants (mathematically, 20 would allow a figure of 45%) and they might have all had a wretched lifestyle. You can beat those odds by having a better-than-average diet & exercise program. You might consider addressing your concerns with your primary healthcare provider and/or a Registered Dietitian.

When the OP responds that he plans on consulting a doctor within the next couple of weeks and already eats well and exercises, the nurse replies by affirming his strategy and expressing support for health responsibility:

I understand. Sounds like you already have a head start on beating the odds. I’m retired from nursing now but I still like seeing people succeed in their own healthcare management. I wish you all the best.

This expert moves away from complete reliance on genetic testing insights to determine health futures, but recognizes that the OP occupies a patient-in-waiting status. As seen in lay community interactions, where posters underscored structural and environmental interactions that contour gene expression, lifestyle interventions were posed as an imperfect but best-possible strategy to manage health uncertainty as patients await more results. Like almost all posters, the exchange between the nurse and the OP brings to light the emphasis on individuals being the mediator and manager of their own health uncertainty in the age of genetic health. The compulsion for individuals to manage their health state through genetic tests discussed on these Reddit posts signals almost an obligation to doing so [5]. As the nurse commends the OP, we see a logic of individualized risk responsibility that rewards health behaviors like diet and exercise. Generally, we observed that experts (though their professional credentials are oftentimes unverifiable) possess more sway and engagement, making them a central character in shaping how data sense-making takes place.

Due to the voluntary nature of Reddit narration, not all diagnostic odysseys come to a legible conclusion. While some posters returned to their threads to share final results of their genetic tests or updates about their condition, most threads are left unresolved. These half-complete health journeys hold valuable clues about the moral and social valuation placed on health and related decision-making, regardless of their outcomes. For lay posters, Reddit provides a support group for those seeking validation or guidance through a deeply personal and scientifically fraught new territory. Ultimately, as patients’ uncertainty about their health states and genetic health results drive the need for more testing, discussions around interpretations, and potential interventions, risk becomes medicalized as a health state. Our data shows the tangible impact DTC genetic health tests have on patient-consumers’ medicalized worldview, their understanding of their risk states, and eventual health management decisions.

## Conclusion

Examining the landscape of genetic health testing as it continues to grow unabated, this article connects two distinct spheres where medical knowledge is produced and contested in the age of genomics: professional conference spaces for experts, and virtual communities for patient-consumers. We show that despite a distinct knowledge gap separating these two groups, both conceptualize genetic risk through an increasingly medicalized lens and exert pressure on the traditional medical establishment to increase focus on the topic of genetic risk.

In sharing advances and discoveries with their colleagues, experts at conferences are stating a case for an idealized future where all disease risk states can be genetically treated. Uncertainty in the present is leveraged by strategically underscoring stories of success and triumph, all while acknowledging the fact that more work needs to be done if medical genomics potential is to be realized. This not only propels the enterprise by calling attention to the need for more institutional resources to be poured into research, but it also galvanizes participants by reminding them of the work ahead. While presenting this future ideal–where all possible disease is predicted and addressed—our conferences ethnographies allowed us to see how experts co-create a vision where present risk-states are necessarily under-treated. Building off of each other’s works and contributions, experts at these conferences remind us that our bodies, having yet to be fully genetically sequenced and analyzed, are inherently and continuously at risk. Such a perspective is then leveraged by experts as a form of motivation to bring in like-minded clinical practitioners who can transform theoretical genetics claims into practical medicalized interventions.

Patient-consumers, like experts, traverse their own version of relating risk to healthiness. One could argue that those who purchased DTC test kits from companies such as 23andme inherited the promises shared by experts (after careful appropriation and packaging by marketeers), believing that genetic health testing can provide answers for health management. That said, for many, the post-test reality is one rife with interpretive troubles and aggravated uncertainty about their health. Without prior training and knowledge, they turn to platforms such as Reddit, where they crowdsource knowledge to help them navigate what to ‘do’ with this newly gained information about themselves and potential risk states. Although the threads examined suggest such crowdsourcing efforts do not provide clear, conclusive answers to diagnostic odysseys (at times confusing them even more), the interactions on these platforms offer both support and a forum to solidify genetic risk as a medically relevant diagnostic state for individuals seeking a framework through which to understand new bodily information. The analysis is summarized below (Table 2).

**Table 2:** Summary of analytical insights.

	Expert (scientists & geneticists) at Conferences	Lay (patient-consumers) on Reddit
**Promise**	An idealized future where disease can be scientifically identified and mitigated	Control can be gained over one’s health outcomes by buying into genetic testing services
**Reality**	An unsettled science relying on uncertainty and generalization to make identifications	Control becomes subject to interpretation via irregular and unstandardized crowd-sourced knowledge

Examining the relationship between genomic information-making and health perceptions, we show how genomic researchers and patient-consumers harness unsettled science to co-produce the medicalization of genetic risk. Scientific experts embrace genetics-based risk assessments and view uncertainty as further reason to improve their craft’s efficacy and accuracy, while simultaneously courting doctors and medical establishments with the promise of medical breakthroughs. On the other side, patient engagement with DTC genetic testing feeds data back into the medical machine, generating more expectations around understanding genomic insights in ways that address health risks. As risk becomes medicalized as a health state necessitating care or treatment in itself, it transforms how society approaches health management and intensifies existing tensions in our biomedicalized landscape. For example, medicalizing risk may magnify pressure on individuals to expend resources in the private health market, as responsible individuals who must manage or ‘treat’ risk consistently (i.e., lifestyle interventions) to stay healthy. Further, given how risk propels the genomic revolution in healthcare, providers may eventually be inclined or expected to treat risk rather than expressed disease. Taken across all fields of medicine, this could substantially alter how healthcare is delivered and exacerbate disparities in access and quality of care. In the larger picture, using risk as a medicalized marker of health may also impact sociocultural values around who is ‘healthy,’ and can precipitate discriminatory insurance coverage, employment, and social stigma toward those with certain risk profiles. If genetic risk becomes akin to sickness, hurdles commonly faced by those struggling with disease may become familiar to those faced with only the potential for disease. Understanding how these online spaces shape peoples’ health, especially as they increasingly seek digital ways to try to make sense of their genetic data, provides valuable insight into how we can better provide clinical care, implement protections and regulations for how DTC tests are delivered, and develop supportive resources like ease of access to genetic counselors via telehealth.

There are a few limitations in this study. For one, while the conferences we attended allowed us an exclusive look into the world of genomics science and knowledge production, our view was limited to the conference attendees who showed up at these events. Other components and actors of the enterprise, including but not limited to research labs or corporations, influence on policy, and marketing are areas of importance that we did not have robust access to. Future research should fill this gap by examining other players and processes tied to genomic knowledge-making. Furthermore, regarding the lay population, we acknowledge that Reddit is a self-selective community. Thus, their attitudes or approaches may not pertain to everyone, especially those who are not as health- and tech-savvy. There remains a larger population of individuals who seek health via other forums (genetic counselors, personal doctors, knowledgeable members in their own networks), and future research should capture these experiences.

## Supporting information

S1 Dataset Clean uncoded posts. Text-based data.
(TXT)

S1 File Python Reddit scraper. Python code.
(PY) Click here for additional data file.

S1 Data (TXT)
